# Biliary Atresia: advances and challenges in early diagnosis and management

**DOI:** 10.1016/j.jped.2026.101522

**Published:** 2026-03-10

**Authors:** Elisa de Carvalho, Mirta Elba Ciocca, Gilda Porta, Natascha Silva Sandy, Carlos Marcelo Timossi, Marcela Godoy, Carola López, Michelle Higuera, Irene Miura, Themis Reverbel Silveira, Fernando Álvarez, Rodrigo Vázquez-Frías, Silvia Filomena Morise, Hector Gustavo Boldrini, Margarita Dolores Ramonet, Miriam Liliana Cuarterolo, Alejandro Costaguta, Rosana Pérez Carusi, Cristina Helena Targa Ferreira, Maria Angela Bellomo-Brandão, Sandra Neri, Marise Elia de Marsillac, Regina Sawamura, Dianora Navarro, Cibele Dantas Ferreira Marques, Juan Juanet Goñi, Lorena Rodríguez González, Humberto E. Soriano, Loreto Hierro Llanillo, Jorge Abdon Bezerra

**Affiliations:** a Hospital da Criança de Brasília, Departamento de Pediatria, Brasília, DF, Brazil; b German Hospital of Buenos Aires, Department of Pediatrics, Buenos Aires, Argentina; c Universidade de São Paulo (USP), São Paulo, SP, Brazil; d Grupo de Hepatologia e Transplante Hepático do Hospital Sírio Libanês e Hospital Menino Jesus, São Paulo, SP, Brazil; e Sociedade Brasileira de Pediatria (SBP), Departamento de Hepatologia, Porto Alegre, RS, Brazil; f Einstein Hospital Israelita, Department of Pediatrics, São Paulo, SP, Brazil; g Miramar MedCom, Department of Research and Development, Mexico City, Mexico; h Hospital Clinico San Borja Arriaran, Pediatric Gastroenterology Unit, Santiago, Chile; i Pereira Rossell Hospital Center, Pediatric Gastroenterology, Hepatology, and Nutrition Service, Montevideo, Uruguay; j Central Hospital of the Armed Forces, National Liver Transplant Program, Montevideo, Uruguay; k National University of Colombia, Faculty of Medicine, Department of Pediatrics, Bogota, Colombia; l El Bosque University, Faculty of Medicine, Bogota, Colombia; m Faculdade de Medicina da Universidade de São Paulo, São Paulo, SP, Brazil; n Hospital Infantil Menino Jesus de São Paulo, Departamento de Pediatria, São Paulo, SP, Brazil; o Hospital Santo Antonio, Porto Alegre, RS, Brazil; p Universidade Federal de Ciências da Saúde de Porto Alegre (UFCSPA), Porto Alegre, RS, Brazil; q Department of Pediatrics, CHU-Sainte Justine, University of Montreal, Quebec, Canada; r Federico Gómez Children's Hospital of Mexico, Research Management Subdirectorate, Mexico City, Mexico; s Latin American Society for Pediatric Gastroenterology, Hepatology, and Nutrition, Mexico City, Mexico; t Alejandro Posadas National Hospital, Department of Pediatrics, Pediatric Gastroenterology Service, Buenos Aires, Argentina; u Italian Hospital of Buenos Aires, Liver Transplant Unit, Pediatric Gastroenterology, Hepatology, and Liver and Intestinal Transplant Service, Buenos Aires, Argentina; v Arenales 1611, Buenos Aires City, Argentina; w Hepatology Department at the Prof. Dr. Juan P. Garrahan Pediatric Hospital, Buenos Aires, Argentina; x Children's Sanatorium, Hepatology and Liver Transplant Unit, Rosario, Santa Fe, Argentina.; y Solid Organ Transplant, El Cruce Hospital, German Hospital, Buenos Aires, Argentina; z Universidade Estadual de Campinas (UNICAMP), Faculdade de Ciências Médicas, Departamento de Pediatria, Campinas, Brasil; aa “JM de los Ríos” Children's Hospital, Caracas, Venezuela; ab Universidade do Estado do Rio de Janeiro, Faculdade de Ciências Médicas, Departamento de Pediatria, Rio de Janeiro, RJ, Brazil; ac Universidade do Estado do Rio de Janeiro, Hospital Universitário Pedro Ernesto, Rio de Janeiro, RJ, Brazil; ad Universidade de São Paulo (USP), Hospital das Clínicas da Faculdade de Medicina de Ribeirão Preto (FMRP), Departamento de Puericultura e Pediatria, Ribeirão Preto, SP, Brazil; ae Central University of Venezuela and Specialist in Pediatric Gastroenterology and Nutrition, Caracas, Venezuela; af Venezuelan Social Security Institute, Dr. Miguel Pérez Carreño General Hospital, Caracas, Venezuela; ag Faculdade de Medicina da Universidade Federal da Bahia (UFBA), Salvador, BA, Braziç; ah Roberto del Río Hospital, Gastroenterology Unit, Santiago, Chile; ai San Juan de Dios Hospital and Clínica Alemana, Gastroenterology-Hepatology Department, Santiago, Chile; aj Pontifical Catholic University of Chile, Department of Gastroenterology and Nutrition, Santiago, Chile; ak La Paz University Hospital, Pediatric Hepatology Service, Madrid, Spain; al University of Texas Southwestern and Children’s Medical Center of Dallas, Department of Pediatrics, Dallas, USA

**Keywords:** Biliary atresia, Neonatal cholestasis, Early diagnosis, Kasai portoenterostomy, Pediatric hepatology

## Abstract

**Objective:**

To review current evidence on early diagnosis and initial management of biliary atresia, highlighting advances achieved in recent decades and the persistent challenges that continue to influence clinical outcomes.

**Sources:**

Relevant literature was identified through structured searches of PubMed and other major biomedical databases, complemented by manual review of reference lists from key original studies, systematic reviews, and position papers addressing biliary atresia and neonatal cholestasis.

**Summary of the findings:**

Biliary atresia remains a highly time-critical disease in pediatric hepatology, with outcomes strongly determined by age at surgical intervention. Population-based screening strategies, particularly stool color card programs, have shown meaningful reductions in age at surgery in selected regions, although their effectiveness is highly dependent on healthcare system organization and follow-up capacity. However, acholic stools remain a valuable sign for clinicians. Laboratory biomarkers such as gamma-glutamyl transferase and matrix metalloproteinase-7 provide complementary diagnostic support. Imaging modalities and liver biopsy continue to play essential roles in the diagnostic work-up. However, several commonly used tests predominantly reflect advanced disease and may be less informative in the earliest stages. Evidence supporting postoperative adjuvant therapies remains heterogeneous, with ongoing controversies regarding their impact on long-term native liver survival.

**Conclusions:**

Despite important advances, early diagnosis of biliary atresia remains challenging in many settings. Integrating clinical vigilance with the judicious use of screening strategies, biomarkers, and diagnostic procedures is essential to optimize outcomes. Continued efforts are needed to refine early diagnostic pathways and to address persistent gaps in evidence, standardization, and access to specialized care.

## Introduction

Biliary atresia (BA) is a progressive fibroinflammatory cholangiopathy of neonatal onset and remains one of the most time‐critical diseases in pediatric hepatology. Recent comprehensive reviews emphasize its complex pathogenesis, heterogeneous presentation, and the central role of early diagnosis in determining outcomes [1]. Although relatively rare, with an incidence ranging from approximately 1 in 5,000 to 1 in 20,000 live births depending on geographic region [[1], [2], [3]], its impact on morbidity, mortality, and health systems is disproportionate. Historically regarded as an invariably fatal cause of neonatal jaundice, the prognosis of BA changed fundamentally with the introduction of Kasai portoenterostomy (KPE) in 1959, which for the first time enabled survival with the native liver [4].

Age at portoenterostomy has consistently emerged as the strongest predictor of native liver survival across diverse national and regional cohorts, with progressively worse outcomes as surgery is delayed [5]. Contemporary cohort studies from different regions have demonstrated substantial heterogeneity in long-term outcomes, largely driven by system-level factors such as early recognition, referral patterns, and centralization of care. Nevertheless, even in highly specialized centers, long-term survival with the native liver is not achieved in most patients. Historically, native liver survival rates ranged from 30–35% at the age of 18 years [6]. Meanwhile, contemporary national cohorts report 20–30-year survival between approximately 25% and 40%, strongly influenced by age at portoenterostomy, centralization of care, and duration of follow-up [7]. In addition, in large European multicenter series, native liver survival has reached approximately 40–55% at 5 years and 35–47% at 10 years in settings where portoenterostomy is performed in more than 90% of affected infants [[8], [9], [10], [11]]. Despite these advances, BA remains one of the leading indications for pediatric liver transplantation worldwide [12]. These data highlight that advances in surgical technique alone are insufficient to meaningfully improve population-level outcomes without parallel progress in early diagnosis and timely referral.

Over the past two decades, major efforts have focused on improving early identification of BA, including the implementation of population-based screening initiatives, refinement of ultrasonographic criteria, and the identification of novel serum biomarkers. While these strategies have contributed to earlier diagnosis in selected settings, delayed recognition remains frequent in many regions, particularly in low- and middle-income countries where access to specialized care and standardized diagnostic pathways may be limited. In addition, the expanding availability of diagnostic modalities has introduced new challenges, as several tests primarily reflect advanced disease changes and may provide false reassurance when applied early in the disease course.

In this context, a critical reappraisal of current approaches to early diagnosis and initial management of BA is warranted. The present review synthesizes contemporary evidence on diagnostic and therapeutic strategies for BA, with a specific focus on early disease. By integrating advances in screening, laboratory biomarkers, imaging, histopathology, and postoperative management, this article aims to critically review current evidence on the early diagnosis and initial management of BA, highlighting which interventions have truly improved outcomes in the 21st century, clarifying the limitations of existing diagnostic tools, and identifying persistent challenges that must be addressed to further optimize care for infants with BA.

## Methods

This narrative review was based on a comprehensive and structured search of the medical literature addressing BA, with particular emphasis on early diagnosis and initial management strategies.

A comprehensive literature search was conducted in MEDLINE (PubMed), EMBASE (Ovid), LILACS, and SciELO. The search strategy incorporated a validated filter to identify randomized controlled trials, systematic reviews, and clinical practice guidelines, and included the following MeSH terms and keywords: (“atresia biliar” [MeSH Terms] OR “biliary atresia” [All Fields]) AND (“diagnosis” [MeSH Terms] OR “diagnosis” [All Fields] OR “early detection” [All Fields]) AND (“tratamiento” [MeSH Terms] OR “treatment” [All Fields] OR “surgery” [All Fields]) AND (Randomized Clinical Trial [ptyp] OR systematic review [sb] OR Clinical Practice Guideline [ptyp]).

Original studies, randomized and non-randomized clinical trials, cohort studies, systematic reviews, and key position papers published primarily in English were considered. Priority was given to studies focusing on neonatal and early infant populations, diagnostic performance, timing of intervention, and clinically meaningful outcomes, such as native liver survival and transplant-free survival. Case reports, studies limited to adult populations, and articles without direct relevance to early disease were excluded. Study design, methodological quality, limitations, and clinical applicability were critically appraised, and areas of consensus, controversy, and unmet research needs were integrated into the narrative synthesis.

### Strategies for early identification of biliary atresia

Early identification of BA remains the most effective strategy to improve long-term outcomes, as it directly influences the timing of surgical intervention and the likelihood of native liver survival. Over the past decades, multiple approaches have been proposed to facilitate earlier recognition of the disease, ranging from population-based screening initiatives to laboratory biomarkers and targeted diagnostic algorithms. Each strategy offers distinct advantages and limitations, particularly when applied during the earliest phases of disease.

#### Population-based screening approaches

Population-based screening using stool color cards has emerged as one of the most impactful public health interventions for early detection of BA. This strategy is based on a simple but highly relevant clinical sign: persistent acholic or hypocholic stools, which reflect impaired bile flow and often precede other manifestations of cholestatic liver disease. Large-scale programs implemented in regions such as Taiwan and Japan have consistently demonstrated that stool color card screening reduces the age at diagnosis and at KPE, with corresponding improvements in jaundice clearance and native liver survival [[13], [14], [15], [16]]. The reported sensitivity of SCC screening ranges between 76% and 90%, while specificity is consistently high, close to 99.9% [17,18]. Notably, comparable reductions in age at portoenterostomy have also been achieved in large population-based cohorts through healthcare system centralization alone, even in the absence of formal population screening programs, highlighting the critical role of organized referral pathways and early specialist access [7].

The strength of stool color card screening lies in its simplicity, low cost, and scalability. It is considered a cost-effective strategy in settings where it has been systematically implemented and evaluated, like Taiwan and Japan. Although the stool color card has been shown to be cost-effective in settings where it has been systematically implemented and evaluated, such as Taiwan and Japan, robust cost-effectiveness data from Europe and low- and middle-income countries remain limited. Implementation requires professional training, integration into primary care workflows, and reinforcement of educational strategies, as the lack of awareness about BA among healthcare providers remains a barrier [13]. And importantly, the effectiveness of stool color card programs is not solely dependent on sensitivity for BA but also on the ability to trigger timely medical evaluation and referral pathways once abnormal stools are identified.

Despite these advantages, stool color card screening is not without limitations. Variability in caregiver interpretation, transient stool color changes, and reduced sensitivity in the earliest neonatal period may lead to false reassurance or delayed recognition in some cases. Additionally, successful implementation requires integration into broader healthcare systems, including education of parents, training of primary care providers, and streamlined referral networks. In regions without structured screening programs, reliance on stool color alone remains inconsistent, contributing to ongoing delays in diagnosis.

In addition to stool color card programs, laboratory-based newborn screening strategies have been evaluated as complementary approaches for early identification of BA. A large multicenter prospective study conducted in the United States enrolled more than 124,000 newborns and applied a two-stage screening protocol based on direct or conjugated bilirubin measurements. This strategy demonstrated excellent diagnostic performance, with a reported sensitivity of 100%, specificity of 99.9%, and negative predictive value of 100% [19]. These findings support the biological and clinical rationale for incorporating early bilirubin assessment into neonatal screening pathways, particularly in healthcare systems where universal laboratory screening is already established. However, implementation requires robust follow-up algorithms, standardized cut-off values, and careful consideration of false-positive rates and resource utilization [19]. Nevertheless, for a clinician confronted with a jaundiced newborn, personal observation of the stools and measurement of direct bilirubin are the most reliable signs for diagnosis of cholestasis and suspicion of BA.

### Laboratory biomarkers

Laboratory biomarkers play a complementary role in the early identification of BA, particularly in infants presenting with persistent neonatal cholestasis. Among routinely available tests, gamma-glutamyl transferase (GGT) has long been recognized as a useful marker of cholangiocyte injury and bile duct obstruction. In many infants with BA, GGT levels are markedly elevated early in the disease course, often exceeding those observed in other causes of neonatal cholestasis. In a prospective study that assessed laboratory biomarkers alongside ultrasound shear-wave elastography, a GGT cutoff > 320 U/L achieved 100% sensitivity, 77.8% specificity, 64.7% positive predictive value, 100% negative predictive value, and an area under the receiver operating characteristic curve (AUROC) of 0.85 for distinguishing BA from other causes of neonatal cholestasis (p < 0.0001) [20]. The diagnostic performance of GGT, however, is influenced by age, disease stage, and overlap with other cholestatic conditions. While high GGT levels may raise suspicion for BA, normal or mildly elevated values do not exclude the diagnosis, particularly in younger infants or in specific phenotypic variants. As such, GGT should be interpreted within a broader clinical and diagnostic context rather than as a standalone discriminator.

More recently, matrix metalloproteinase-7 (MMP-7) has gained attention as a promising biomarker for BA. MMP-7 is predominantly expressed by cholangiocytes and is involved in extracellular matrix remodeling, epithelial injury, and fibrogenesis – processes that are central to the pathophysiology of BA. Multiple studies have demonstrated high diagnostic accuracy of serum MMP-7 for distinguishing BA from other causes of neonatal cholestasis, even at early stages of disease. A network meta-analysis of 40 studies further supported a pooled sensitivity of 91.5% (95% CI 0.893–0.934) and specificity of 84.3% (95% CI 0.820–0.863) [21].

A systematic review and meta-analysis identified GGT, together with MMP-7 and IL-33, as relevant biomarkers for both the diagnosis of BA and prognosis after KPE. For GGT alone, pooled diagnostic performance showed a sensitivity of 80%, specificity of 79%, and an AUROC of 0.96. When combined with other biomarkers, diagnostic accuracy improved further, with MMP-7 demonstrating a sensitivity of 96%, specificity of 91%, and an AUROC of 0.98 [22]. Increased serum levels of MMP-7 also correlate with the severity of fibrosis and the need for transplantation, highlighting its role not only as a diagnostic tool but also as a prognostic biomarker in BA [23].

Despite its strong biological plausibility and high diagnostic accuracy, the clinical adoption of MMP-7 remains limited by availability, assay standardization, and cost, and it currently represents a valuable adjunctive biomarker rather than a population-level screening tool. However, reported diagnostic cut-off values for MMP-7 vary substantially across studies, reflecting differences in assay platforms, patient age, disease stage, and study design. Recent large validation studies highlight the need for standardized thresholds and prospective external validation before widespread clinical implementation [24]. Evidence also supports an incremental benefit when laboratory markers are combined with imaging modalities. For example, the combination of GGT with ultrasound findings significantly improves discrimination between BA and non-BA cholestasis compared with either method alone [25].

### Ultrasonography as first-line imaging

Imaging studies are central to the evaluation of suspected BA, but their diagnostic yield varies with disease stage, timing, and expertise, making integration with clinical and laboratory data essential to avoid diagnostic delay.

Ultrasonography is a noninvasive, radiation-free, real-time modality and is widely used as the first-line imaging tool in the evaluation of infants with suspected BA [26,27]. The most robust and consistently validated sonographic markers are gallbladder abnormalities and the triangular cord sign (TCS), which, when interpreted together, achieve the highest diagnostic accuracy [28].

Gallbladder abnormalities include no visualization of the lumen, reduced size (typically < 15–19 mm), abnormal shape, wall thickening or irregularity, and lack of postprandial contraction. Absence of the gallbladder is highly specific for BA, with reported specificity of up to 98–100%, but sensitivity is limited, as a gallbladder structure may still be present in some affected infants. In contrast, a small or morphologically abnormal gallbladder is more sensitive but less specific [29]. Diagnostic accuracy improves when gallbladder morphology is incorporated into classification systems or dimensional ratios, particularly when combined with the TCS [30].

The triangular cord sign corresponds to echogenic periportal thickening (> 3–4 mm) anterior to the right portal vein, reflecting fibrotic remnants of the extrahepatic bile duct. It represents the most specific single ultrasonographic feature of BA, with specificity consistently reported between 95% and 100%, although sensitivity varies widely (approximately 49–85%), especially in infants younger than 30 days [31,32]. An updated meta-analysis reported a pooled sensitivity of approximately 80–85% and specificity approaching 96–100% [33].

When gallbladder abnormalities and the TCS are assessed in combination, overall diagnostic accuracy increases substantially, reaching values close to 95–98% across studies [28,29]. Additional supportive findings—such as no visualization of the common bile duct, enlargement of the hepatic artery, subcapsular hepatic flow on Doppler imaging, and indirect signs of chronic liver disease or portal hypertension—may further raise suspicion but are less specific when considered in isolation [34]. The detection of polysplenia and pre-duodenal portal vein leads to suspect a BA.

Increased hepatic subcapsular flow is more commonly observed in the context of established fibrosis and portal hypertension and reflects a compensatory response to altered intrahepatic hemodynamics [[35], [36], [37]]. For this reason, hepatic subcapsular flow is more reflective of established disease and has limited value for early diagnosis of BA. Finally, it is important to emphasize that a normal gallbladder or absent triangular cord sign does not rule out BA, particularly in the earliest stages of disease, reinforcing the need to integrate ultrasonographic findings with clinical and laboratory.

#### Liver biopsy

Liver biopsy remains an established component of the diagnostic evaluation of persistent neonatal cholestasis and is widely used when BA is suspected [26]. Percutaneous liver biopsy is the preferred approach in most centers; however, it should be incorporated into diagnostic pathways in a manner that does not delay surgical exploration when clinical suspicion is high [38,39].

When interpreted by experienced pathologists, liver biopsy distinguishes obstructive cholangiopathies such as BA from intrahepatic causes of cholestasis in approximately 90–95% of cases [26,40]. Reported sensitivity varies from 60% to 95%, depending on patient age and histopathologic expertise [41]. Typical histologic features include ductular proliferation, bile plugs, portal edema, and portal fibrosis, usually with preservation of lobular architecture. However, histologic findings may be heterogeneous, particularly early in the disease course.

In the multicenter ChiLDReN Consortium study of 227 biopsies, liver biopsy demonstrated a sensitivity of 88% and a specificity of 90.1%; notably, ductular proliferation was absent in 22.8%, and bile plugs in 25% of confirmed BA cases, underscoring the variability of early histologic presentation [40]. Sequential biopsy studies further illustrate the temporal evolution of histologic features, which are frequently absent around 40 days of age but become more consistently detectable after 60 days [42].

Meta-analyses confirm high overall diagnostic performance, with sensitivity ranging from 88% to 96% and specificity from 91% to 100%, with the highest yield reported in infants ≤60 days of age, in whom both sensitivity and specificity exceed 95% [41]. Although invasive, major complications are uncommon, occurring in approximately 0.5–4% of procedures, particularly in the presence of coagulopathy, very young age, or advanced liver disease [43,44,45].

Overall, liver biopsy provides valuable diagnostic and prognostic information in BA, particularly when noninvasive methods are inconclusive. Its limitations in very early disease highlight the importance of interpreting histologic findings within an integrated clinical, laboratory, and imaging framework, and of embedding biopsy into diagnostic pathways that prioritize timely surgical intervention.

#### Intraoperative cholangiography

Intraoperative cholangiography is widely regarded as the reference standard for establishing or excluding BA in infants with persistent cholestasis. It is typically performed as the final diagnostic step immediately before KPE, particularly when noninvasive investigations and liver biopsy do not provide a definitive diagnosis, but clinical suspicion remains high. By directly visualizing the extrahepatic biliary tree, intraoperative cholangiography allows real-time surgical decision-making and, in many centers, is performed using minimally invasive approaches that permit portoenterostomy during the same operative session when BA is confirmed [26,38,46,47].

Accurate interpretation of intraoperative cholangiography is critical, as several non–BA cholestatic disorders may be associated with hypoplastic or poorly opacified bile ducts. Alagille syndrome, neonatal sclerosing cholangitis, and, less frequently, cystic fibrosis are among the conditions most commonly reported to mimic the cholangiographic appearance of BA, increasing the risk of false-positive interpretation [26,38,46,47]. In addition, some patients with these pathologies frequently show transient acholic stools. Preoperative clinical and genetic evaluation is therefore essential to contextualize intraoperative findings and reduce diagnostic error. Importantly, multiple studies have demonstrated unfavorable outcomes when KPE is performed in infants with Alagille syndrome [48,49]. These observations were reinforced by the multinational GALA study, which confirmed poorer outcomes in this population, further emphasizing the need to avoid portoenterostomy when an alternative diagnosis is likely [50].

Taken together, these data underscore the importance of expert interpretation of intraoperative cholangiography within an integrated diagnostic framework that incorporates clinical features, preoperative investigations, and genetic considerations, thereby minimizing unnecessary surgical intervention and its associated risks.

### Integrating clinical suspicion and diagnostic pathways

Early diagnosis of BA depends on clinical vigilance, timely laboratory evaluation, and access to specialized diagnostic pathways. Persistent jaundice with pale stools or conjugated hyperbilirubinemia warrants prompt assessment. Diagnostic findings must be interpreted in relation to disease stage, and system-level factors remain key determinants of timely diagnosis.

### Diagnostic tests of limited clinical value: high cost, invasiveness, poor accuracy, or delayed findings

Several diagnostic modalities have been explored in the evaluation of neonatal cholestasis; however, many have limitations for early identification of biliary atresia due to high cost, invasiveness, low diagnostic accuracy, or reflection of advanced disease stages. These include hepatobiliary scintigraphy, elastography, magnetic resonance cholangiopancreatography (MRCP), endoscopic retrograde cholangiopancreatography (ERCP), and percutaneous cholecysto-cholangiography (PCC).

#### Hepatobiliary scintigraphy

Hepatobiliary scintigraphy (HBS) using Tc-99m–labeled iminodiacetic acid derivatives has historically been used to assess biliary patency in cholestatic infants [26,27]. Although reported sensitivity is high—approaching 98–99% in meta-analyses—specificity is consistently limited, typically ranging from 45% to 75% [51]. Absent tracer excretion may also occur in several intrahepatic cholestatic disorders, including idiopathic neonatal hepatitis, interlobular bile duct paucity, and parenteral nutrition–associated cholestasis, resulting in frequent false-positive interpretations [52,53]. In a comparative study, HBS achieved 88% sensitivity but only 46% specificity, with an overall accuracy of 67%, whereas liver biopsy demonstrated superior performance (100% sensitivity, 94% specificity, and 97% accuracy) [54].

#### Elastography

Elastography assesses liver stiffness, which primarily reflects fibrosis and, at later stages, portal hypertension. In BA, stiffness values are consistently higher than in other cholestatic conditions; however, this difference largely reflects progressive structural injury rather than the earliest stages of disease [20,55].

Individual studies report moderate to high diagnostic accuracy, with marked age dependency. In a multicenter cohort, transient elastography showed excellent performance in infants ≤ 90 days, yet stiffness closely correlated with histologic fibrosis [56]. Similarly, shear-wave elastography demonstrated an AUROC of 0.82 in infants ≤ 45 days, with improved performance in older infants [57]. Meta-analyses report pooled sensitivities of 77–86% and specificities of 79–86%, with accuracy increasing as fibrosis advances, but inferior to highly specific grayscale ultrasound markers such as the triangular cord sign or gallbladder abnormalities [30,58].

Overall, elastography provides complementary information but has limited value for early diagnosis, given its dependence on disease progression and overlap with other inflammatory or cholestatic conditions.

#### Magnetic resonance cholangiopancreatography

MRCP has been evaluated as a noninvasive method to visualize the extrahepatic biliary tree, typically relying on no visualization of ducts or gallbladder abnormalities [59]. However, diagnostic performance is limited in neonates and young infants. In a pilot study, extrahepatic bile ducts were visualized in only 50% of infants younger than 30 days and 62.5% of those older than 3 months [60].

Meta-analyses demonstrate relatively high sensitivity but consistently low specificity, often below 40%, leading to frequent false-positive results [53]. In a prospective cohort, three-dimensional MRCP achieved sensitivity near 99% but specificity of only 36% [61]. Technical constraints, including small duct caliber, motion artifacts, frequent need for sedation, cost, and limited availability, further restrict its role in early diagnostic pathways [26,27].

### Endoscopic retrograde cholangiopancreatography

ERCP has been employed in selected tertiary centers to assess extrahepatic duct patency in cholestatic infants [62]. In a single-center series spanning 14 years, ERCP demonstrated sensitivity of 86–100%, specificity of 79–94%, and negative predictive value of 100% for excluding BA [63]. Complete visualization of the biliary tree or bile flow at the papilla effectively ruled out the diagnosis.

Despite favorable accuracy in expert hands, ERCP in infants is invasive, requires deep sedation or general anesthesia, and depends on specialized equipment and operator expertise that are not widely available. Adverse events, including post-ERCP pancreatitis, bleeding, and perforation, further limit its practicality. Consequently, ERCP is best reserved for highly selected, equivocal cases rather than incorporated into early diagnostic algorithms [26,64].

### Percutaneous cholecysto-cholangiography

PCC has demonstrated high diagnostic performance in selected cohorts. A network meta-analysis estimated sensitivity near 100% and specificity of approximately 87%, placing PCC among the better-performing modalities in specialized settings [21]. Earlier series suggested that PCC may exclude BA when a well-visualized, dilated gallbladder is present, potentially reducing negative laparotomies [65,66]. More recent prospective studies combining PCC with microbubble contrast and liver biopsy have reported high diagnostic yield with mostly minor complications [67].

Nevertheless, routine early use of PCC is constrained by invasiveness, need for sedation, limited availability of pediatric interventional radiology, and the risk of incomplete delineation in proximal obstructions — often necessitating subsequent surgical cholangiography. Current diagnostic pathways therefore, reserve PCC for highly selected scenarios rather than routine early evaluation [26].

### Treatment: evidence, timing, and controversies

#### Timing of Kasai portoenterostomy

Early surgical intervention with KPE remains the single most important determinant of native liver survival in BA. Across multiple cohorts and registries, age at surgery consistently emerges as the strongest prognostic factor. A meta-analysis including nine large cohorts demonstrated that KPE performed early—particularly within the first 30 days of life—was associated with significantly improved native liver survival at 5, 10, and even 20 years, whereas later surgery was linked to a more than twofold increased hazard for liver transplantation (hazard ratio 2.12) [68].

These findings are supported by data from the Western Pediatric Surgery Research Consortium, which estimated a 2% decline in transplant-free survival for each additional day of surgical delay [69]. Although the traditional “60-day dogma” has been challenged, outcomes remain consistently superior when surgery is performed before 30–45 days [70]. Registry data reinforce this time dependency: in the Japanese national registry, 25-year native liver survival was 35% among infants operated before 31 days compared with 25% in those treated later [71]. Similar trends were observed in a large European cohort of more than 1,400 patients, in which earlier surgery was associated with higher rates of jaundice clearance [8].

Despite widespread recognition of BA as a surgical emergency, delays in diagnosis and referral persist globally. It has been well known for decades that BA is a surgical emergency. More recently, Lacaille and colleagues reinforced in a Delphi consensus that reducing time to diagnosis remains a major objective of BA care, as delays directly compromise outcomes [72]. Davenport et al*.* provided evidence that centralization of BA care in specialized centers leads to earlier referral, lower median age at surgery, and improved postoperative outcomes [7]. Population-based studies confirm disparities in timing and prognosis across regions. A Saudi Arabian national study (2000–2018) reported that the median age at KPE remained above the optimal threshold, with native liver survival significantly lower compared to countries with centralized care systems [73]. Similarly, US registry data indicate that many children still undergo surgery after 60 days, highlighting the urgent need for public health strategies to improve early recognition and referral [74]. Reports from resource-limited settings also confirm that even in constrained systems, earlier surgery translates into better outcomes [75].

Despite early KPE improving transplant-free survival, long-term follow-up shows that complications such as portal hypertension and cholangitis remain common, requiring lifelong monitoring [76]. Clearance of jaundice within three months postoperatively is a strong predictor of 5-year native liver survival [77]. Isolated reports of extremely early KPE (within the first 2 weeks of life) demonstrate dramatic improvements in prognosis, including normalization of liver function and avoidance of early transplantation [78,79].

#### Cholangitis and short-term antibiotic prophylaxis

Cholangitis is the most frequent complication following KPE, affecting approximately 40–93% of children with BA, with most episodes occurring within the first two years of life [80,81]. Both early and recurrent cholangitis have been consistently associated with poorer jaundice-free and native liver survival. Diagnostic definitions usually combine clinical features — such as fever, worsening jaundice, acholic stools, and abdominal discomfort—with laboratory and imaging findings, including inflammatory markers, rising transaminases, bilirubin, gamma-glutamyl transferase, or bile lakes [80].

The rationale for short-term antibiotic prophylaxis in the immediate postoperative period is to prevent early cholangitis, a complication strongly linked to adverse outcomes. Oral regimens such as trimethoprim–sulfamethoxazole or neomycin administered for 6–12 months have been used as primary or secondary prophylaxis in some centers [82,83]. However, potential benefits must be weighed against the risk of selecting resistant organisms and severe infections. Empirical choices have evolved over time, shifting from third-generation cephalosporins toward broader-spectrum agents in resistant cases [83]. Regional data indicate that gram-positive organisms, particularly *Enterococcus* species, may account for nearly 50% of late cholangitis episodes, further complicating empirical strategies [84].

Despite widespread clinical use, evidence supporting routine prophylaxis remains conflicting. A systematic review including 714 patients found no significant reduction in cholangitis incidence or improvement in native liver survival [82]. These findings were confirmed by subsequent meta-analyses [85]. Randomized controlled data are limited; one trial showed reduced early-onset cholangitis with prolonged intravenous antibiotics but no improvement in jaundice clearance or long-term survival [86]. Retrospective studies have similarly failed to demonstrate consistent benefit [[87], [88], [89]]. Overall, while short-term prophylaxis may reduce very early cholangitis in selected settings, robust evidence supporting routine use in all patients is lacking.

#### Ursodeoxycholic acid

Off-label use of ursodeoxycholic acid (UDCA) following KPE is routine in many centers worldwide, with surveys from Europe and Japan reporting near-universal adoption [90,91]. Proposed mechanisms include modulation of bile acid composition, stabilization of hepatocyte membranes, stimulation of bicarbonate-rich hypercholeresis, cholangiocyte protection, and immunomodulatory effects [91].

Clinical data regarding UDCA after KPE suggest biochemical benefit but remain inconclusive with respect to long-term clinical outcomes. A crossover withdrawal study demonstrated deterioration in liver biochemistry following UDCA discontinuation, with subsequent improvement upon reintroduction, supporting a pharmacologic effect on bile flow and cholestasis markers [92]. In contrast, concerns regarding potential harm have largely been derived from experimental or mechanistic hypotheses rather than robust clinical evidence. While molecular studies have suggested theoretical adverse effects of prolonged bile acid exposure, clinically significant toxicity attributable to UDCA has not been consistently reported in pediatric BA cohorts [93]. More recent multicenter observational data indicate that low-dose UDCA (≤ 10 mg/kg/day), particularly during the first year after KPE, may be associated with improved bilirubin and bile acid profiles, whereas prolonged use beyond 12–36 months does not appear to confer additional benefit [94].

Systematic reviews in pediatric cholestasis consistently report improvement in liver biochemistry and pruritus, but no definitive effect on native liver survival [95]. Combination strategies with corticosteroids may accelerate early jaundice clearance, although the independent contribution of UDCA is unclear [96]. Importantly, in cases of Kasai failure, continuation of UDCA is generally discouraged due to the risk of bile acid accumulation and complications [97,98].

Taken together, UDCA remains widely used as supportive therapy after KPE, with a favorable safety profile and biochemical benefits, although high-quality evidence demonstrating improvement in long-term transplant-free survival is lacking.

#### Phenobarbital

Phenobarbital has historically been used as a choleretic agent in infants with cholestasis, including those undergoing KPE. However, available evidence does not support its routine postoperative use. Randomized trials failed to demonstrate clinical benefit [99,100], and comparative studies showed limited efficacy in reducing direct bilirubin, with no improvement in outcomes compared to UDCA [101].

Earlier reports suggesting benefit as part of combination regimens likely reflected the effects of corticosteroids or UDCA rather than phenobarbital itself [102]. In the absence of demonstrated efficacy and given concerns regarding potential neurotoxicity in early life, phenobarbital has no established role in the postoperative management of BA.

#### N-acetylcysteine

N-acetylcysteine has been explored as an adjunctive therapy after KPE due to its antioxidant and hepatoprotective properties and its ability to stimulate glutathione synthesis and bile flow [99]. However, current evidence does not support its routine use [103]. Although generally well tolerated, the lack of efficacy precludes its incorporation into routine postoperative care. Broader perioperative literature similarly fails to demonstrate consistent benefit [[104], [105], [106]]. At present, N-acetylcysteine should be considered investigational and not part of routine clinical care

#### Corticosteroids

Inflammation has long been implicated in the pathogenesis of BA, leading to the hypothesis that corticosteroids could enhance bile drainage after KPE. Nevertheless, the role of adjuvant corticosteroid therapy following Kasai portoenterostomy for biliary atresia remains a subject of ongoing debate.

The multicenter Steroids in Biliary Atresia Randomized Trial (START) demonstrated that high-dose corticosteroids (intravenous methylprednisolone 4 mg/kg/day for 2 weeks followed by oral prednisolone 2 mg/kg/day for 2 weeks with subsequent tapering over 9 weeks) initiated within 72 hours of hepatoportoenterostomy failed to significantly improve bile drainage at 6 months post KPE (58.6% versus 48.6% in placebo group; adjusted relative risk 1.14, 95% confidence interval 0.83-1.57; p = 0.43) or transplant-free survival at 24 months of age (58.7% versus 59.4%; adjusted hazard ratio 1.0, 95% confidence interval 0.6-1.8; p = 0.99). Moreover, steroid therapy was associated with an earlier onset of serious adverse events by 30 days post-KPE (37.2% versus 19.0%; p = 0.008), including complications at surgical anastomoses and intestinal perforation [107].

A subsequent Cochrane systematic review and meta-analysis corroborated these findings, concluding that glucocorticosteroids after Kasai portoenterostomy do not result in statistically significant treatment differences in bile drainage at 6 months, although a small clinical benefit could not be excluded [108].

More recent meta-analyses have suggested potential benefits in specific subgroups, particularly infants undergoing Kasai portoenterostomy at less than 70 days of age, with improved jaundice clearance rates at 6, 12, and 24 months (risk ratio 1.35, 95% confidence interval 1.18-1.55; p < 0.001 at 6 months) and improved native liver survival at 24 months (risk ratio 1.31, 95% confidence interval 1.03-1.68; p = 0.028), though short-term native liver survival and cholangitis incidence remained unaffected [109].

Newer strategies, including rectal budesonide, remain experimental and require further validation [110]. Steroid pulse therapy has also been proposed for infants with poor postoperative reduction in bilirubin levels, although supporting data are limited [111].

The heterogeneity in steroid regimens, dosing protocols, and patient populations across studies continues to complicate definitive conclusions, though the preponderance of high-quality evidence does not support routine adjuvant corticosteroid use in the immediate postoperative period following Kasai portoenterostomy for biliary atresia [1,[112], [113], [114]].

Overall, corticosteroids are not used routinely after KPE but may be considered in carefully selected and well-evaluated cases, particularly in younger infants or those with suboptimal early bile drainage.

### Long-term antibiotic prophylaxis after Kasai portoenterostomy

The rationale for long-term antibiotic prophylaxis after KPE is grounded in the well-established association between recurrent bacterial cholangitis, progressive fibrosis, disease progression, and the subsequent need for liver transplantation [115]. Cholangitis occurs most frequently within the first two postoperative years, although late episodes are also reported, making its prevention and early recognition a central component of long-term management. However, the absence of a standardized definition of cholangitis, low rates of pathogen isolation, and heterogeneity in reported outcomes substantially limit the interpretation of available data [80].

Multiple studies and systematic reviews have assessed the effectiveness of prolonged antibiotic prophylaxis with conflicting results. A meta-analysis including more than 700 patients found no significant reduction in the incidence of cholangitis or improvement in native liver survival among patients receiving prophylaxis [116]. Similarly, a multicenter retrospective study demonstrated no difference in cholangitis rates between children treated with prophylactic antibiotics and those who were not; notably, prophylaxis was associated with an earlier onset of cholangitis episodes [89]. A prospective cohort study likewise failed to show meaningful improvement in clinical outcomes with routine prophylaxis [88].

In contrast, smaller studies and selected cohorts have suggested potential benefit. A randomized controlled trial comparing short versus prolonged courses of intravenous antibiotics demonstrated a reduction in early-onset cholangitis with longer treatment duration, although this did not translate into improved native liver survival [86]. Earlier series also reported fewer cholangitis episodes with oral trimethoprim–sulfamethoxazole or neomycin [117]. More aggressive regimens combining broad-spectrum antibiotics with immunoglobulin have shown marked reductions in early cholangitis, but raise important concerns regarding generalizability, antimicrobial stewardship, and the risk of selecting resistant organisms [118].

A systematic review synthesizing these data concluded that the overall evidence remains inconclusive, with substantial heterogeneity across studies and no clear demonstration of long-term benefit [82]. Taken together, current literature does not support the routine use of long-term antibiotic prophylaxis for all patients after KPE. The balance between a potential reduction in recurrent cholangitis and the risk of antimicrobial resistance remains unresolved, underscoring the need for well-designed prospective randomized trials to better define its role in long-term management.

### Conclusions and future directions

BA remains a paradigmatic example of a time‐critical disease in pediatric hepatology, in which outcomes are determined less by the availability of advanced therapies and more by the timeliness and coherence of diagnostic and surgical pathways. Over the past decades, substantial progress has been made in understanding the biological basis of early disease and in refining diagnostic strategies, particularly through population-based screening initiatives, targeted ultrasonographic markers, and the selective use of laboratory and histologic tools. Nevertheless, delayed diagnosis continues to be common in many settings, limiting the impact of these advances on population-level outcomes.

Timely KPE remains the cornerstone of treatment and the most powerful modifiable determinant of native liver survival. In contrast, the evidence supporting adjuvant medical therapies remains heterogeneous, with few interventions demonstrating consistent long-term benefit. This reinforces the central role of early recognition, structured referral networks, and experienced multidisciplinary care, rather than reliance on postoperative pharmacologic strategies to compensate for delayed intervention.

Looking ahead, future progress in BA will depend on further shifting diagnosis toward the earliest weeks of life. Expansion of population-based screening programs, broader validation of promising biomarkers such as matrix metalloproteinase-7 across diverse healthcare settings, and integration of genetic evaluation into early diagnostic algorithms represent key priorities. At the same time, prospective studies are needed to better define which subgroups may benefit from targeted adjuvant therapies and to identify novel antifibrotic or disease-modifying approaches.

Ultimately, improving outcomes in BA will require coordinated efforts that align biological insight with pragmatic clinical pathways, health system organization, and public health strategies. Only through such integrated approaches can the full potential of early diagnosis and timely intervention be realized for affected infants.

## Funding sources

None.

## Data availability statement

The data that support the findings of this study are available from the corresponding author.

## Conflicts of interest

The authors declare no conflicts of interest.
